# Genomic meta-analysis of growth factor and integrin pathways in chronic kidney transplant injury

**DOI:** 10.1186/1471-2164-14-275

**Published:** 2013-04-23

**Authors:** Amrita Dosanjh, Elizabeth Robison, Tony Mondala, Steven R Head, Daniel R Salomon, Sunil M Kurian

**Affiliations:** 1 Department of Molecular and Experimental Medicine, The Scripps Research Institute, La Jolla, CA, 92037, USA

**Keywords:** Chronic rejection, NA Microarrays, Genomics, Growth factors, Integrins, Proteomics, Meta-analysis, Chronic Allograft Nephropathy /Tubular atrophy with interstitial fibrosis (CAN/IFTA), Kidney transplantation

## Abstract

**Background:**

Chronic Allograft Nephropathy (CAN) is a clinical entity of progressive kidney transplant injury. The defining histology is tubular atrophy with interstitial fibrosis (IFTA). Using a meta-analysis of microarrays from 84 kidney transplant biopsies, we revealed growth factor and integrin adhesion molecule pathways differentially expressed and correlated with histological progression. A bioinformatics approach mining independent datasets leverages new and existing data to identify correlative changes in integrin and growth factor signaling pathways.

**Results:**

Analysis of CAN/IFTA Banff grades showed that hepatocyte growth factor (HGF), and epidermal growth factor (EGF) pathways are significantly differentially expressed in all classes of CAN/IFTA. MAPK-dependent pathways were also significant. However, the TGFβ pathways, albeit present, failed to differentiate CAN/IFTA progression. The integrin subunits β8, αv, αμ and β5 are differentially expressed, but β1, β6 and α6 specifically correlate with progression of chronic injury**.** Results were validated using our published proteomic profiling of CAN/IFTA.

**Conclusions:**

CAN/IFTA with chronic kidney injury is characterized by expression of distinct growth factors and specific integrin adhesion molecules as well as their canonical signaling pathways. Drug target mapping suggests several novel candidates for the next generation of therapeutics to prevent or treat progressive transplant dysfunction with interstitial fibrosis.

## Background

Chronic Allograft Nephropathy (CAN) is defined as a clinical entity by chronic kidney injury leading to progressive loss of kidney transplant function in the absence of another known cause. In histological terms, the findings on a biopsy that match this clinical presentation are interstitial fibrosis and tubular atrophy (IFTA) based on the Banff ’05 classification [1]. We will refer to this entity as CAN/IFTA to respect both the clinical and pathological definitions. More recently, several groups have observed that histological IFTA with interstitial inflammation identifies the patients at highest risk for graft loss [2-5]. It is suggested that this latter presentation, IFTA/inflammation is a workable definition of chronic rejection. It remains uncertain whether all CAN/IFTA with progressive kidney dysfunction is a consequence of immune-mediated chronic rejection or whether there are yet additional mechanisms to discover.

Grading IFTA histology is a means of expressing the severity of chronic kidney injury. Fibrosis represents the end result of kidney tissue injury, which includes the donor organ’s history, impact of ischemia/reperfusion, alloimmune-activated T and B cell-mediated, antibody-mediated and inflammation-mediated vascular injury [6-8]. Regardless of the proximate causes, interactions between growth factors and integrin adhesion molecules are important mechanisms in the cycle of injury, repair and fibrosis that are also widely accepted as downstream events in CAN/IFTA [8-13].

The goals of the current study were a meta-analysis and the mapping of functional pathways using gene expression array data generated in our own laboratory as well as two independent, external datasets (total 84 kidney transplant biopsies). The general aim of a meta-analysis is to more powerfully estimate the true “effect size” than possible with a single study. There have been other studies that have looked at the gene expression in CAN/IFTA using microarrays. In one study, two independent CAN/IFTA data sets from the public domain (22 and 14 biopsies, respectively) were used to identify 309 genes that were differentially expressed in CAN/IFTA and were tested as predictors of CAN/IFTA on an additional set of 6 biopsies [14]. By applying Fisher’s exact test to identify enriched KEGG pathways, 6 different KEGG pathways, all metabolism related, were over-represented. A second study of 24 IF/TA biopsies showed that differentially expressed genes were associated with immune response, inflammation, and matrix deposition [15]. Another study looked at gene expression in CAN/IFTA biopsies describing “pathogenesis-based transcript sets” for injured/diseased tissue [16]. Profiling of 150 kidney transplant biopsies to study the impact of inflammation with fibrosis, showed that Toll-like receptor signaling, antigen presentation/dendritic cell maturation, IFN-gamma-inducible response and cytotoxic T lymphocyte-associated changes were associated with inflammatory changes [4].

In the current study we focused on identifying the differentially expressed growth factor and integrin signaling pathways since there has not been any previous meta-analysis that has focused on these mechanisms despite literature implicating individual growth factors and integrins with CAN/IFTA. Our study covered biopsies representative of the early development of CAN/IFTA (Banff grade 1) and the subsequent progression to moderate and severe forms (Banff grades 2 and 3). These new results identify specific growth factor and integrin pathways that allowed us to map a number of potentially novel and drug-able targets.

## Results and discussion

There have been meta-analyses in kidney transplantation, which have been published with specific focuses. The meta-analysis done by Park et al., [17] focused on global profiling using GEO datasets on a broad term called “kidney rejection” that included acute as well as chronic rejection. A similar study that analyzed acute kidney rejection, also used a small number of CAN (n = 7) samples and looked at a predefined subset of acute rejection transcripts (ARTS) and pathogenesis based transcripts (PBTs) in these rejection types [18]. Finally another study, again primarily of acute renal allograft rejection, looked at the expression of metzincins (METS), and metzincins and related genes (MARGS) in renal allograft biopsies using four independent microarray data sets [19]. Their data sets also included a small group (n = 7) of IFTA samples.

In the current study we specifically wanted to test the hypothesis that a meta-analysis of CAN/IFTA samples from different sources would confirm the role of specific growth factors and/or integrin pathways and potentially reveal new pathways correlative with the progression of CAN/IFTA, which individual studies failed to identify due to the limitations with small sample sizes. We mapped the differentially expressed genes in the varying grades of CAN/IFTA to molecular pathways with a special emphasis on established growth factor and integrin signaling pathways.

### Growth factor pathway signaling

In the first comparison of Banff 0 (n = 25) vs. All CAN/IFTA (Banff 1, 2&3; n = 59), class comparisons yielded 4683 differentially expressed probesets. The differential expression and directionality of fold changes is mentioned for each class comparison in Additional file 1: Tables S1, Additional file 2: Table S2, Additional file 3: Table S3 and Additional file 4: Table S4 for all the results discussed below. Specifically, IPA identified that these 4683 genes fall into 123 significant pathways (SD1). When we applied a Benjamini Hochberg correction for multiple testing to the pathway analysis, there were 74 pathways that were significant (p < 0.05). In this comparison HGF, VEGF, Epidermal Growth Factor (EGF), Insulin-like Growth Factor 1 (IGF-1), TGF-beta and Platelet-derived Growth Factor (PDGF) signaling pathways were all significantly differentially expressed, even with multiple testing correction.

The second comparison was Banff 0 (n = 25) vs. Banff 1(n = 14) (mild CAN/IFTA), which yielded 1430 significantly differentially expressed probesets and 84 significant pathways (SD2). HGF, PDGF, VEGF and EGF signaling pathways were differentially expressed as in the comparison to All CAN/IFTA samples. However, Fibroblast Growth Factor (FGF) was also found but the TGFβ pathway was not significantly expressed.

The third comparison, Banff 0 (n = 25) vs. Banff 2&3 (n = 29) (moderate/severe CAN/IFTA), yielded 1978 significantly differentially expressed probesets and 58 significant pathways (SD3). The EGF, FGF, HGF and IGF-1 pathways were all significant. Again, the TGFβ pathway was not significantly differentially expressed in this analysis. Finally, we compared mild to moderate/severe CAN/IFTA (Banff 1; n = 25 vs. Banff 2&3; n = 29) and revealed 1362 significantly differentially expressed genes and 44 significant pathways total (SD4). Of 44 pathways identified, there was only one growth factor pathway, IGF-1, with 7 DE genes (p = 0.05; 5/7 DE genes were upregulated in moderate/severe CAN/IFTA). However, it is important to note here that the FDR ranges for this particular class comparison ranged from 14-19%, a much less robust gene expression profile statistically than any of the other comparisons performed. Given that everything else was technically the same for all comparisons, we believe these results reflect the biology and demonstrate that the differences between early and more advanced CAN/IFTA as judged by non-quantitative histology in these studies were significantly less. It is possible that as quantitative digital histology becomes more widely available [20] that future comparisons of early to more advanced CAN/IFTA will be more robust.

In sum, the HGF and FGF signaling pathways were significantly upregulated in early CAN/IFTA (Banff 0 vs. 1) and continued to be upregulated in moderate/severe CAN/IFTA (Banff 0 vs. Banff 2&3). In contrast, it was observed that the VEGF and PDGF signaling pathways were only significantly upregulated in early CAN/IFTA compared to Banff 0 biopsies and IGF1 was the only significant growth factor pathway upregulated in moderate/severe CAN/IFTA. Table 1 summarizes all the pathways in these four different comparisons.

**Table 1:** Growth factor signaling pathways significantly differentially expressed between the Banff grades of CAN/IFTA

**Normals vs. CAN (Banff 1,2 &3)**	**p-value**
HGF Signaling^$^	0.0001
VEGF Signaling*	0.0016
EGF Signaling^$^	0.0017
IGF-1 Signaling*	0.0022
TGF-β Signaling	0.0056
PDGF Signaling*	0.0112
**CAN Banff 0 vs. Banff 1**	**p-value**
HGF Signaling^$^	0.0011
FGF Signaling*	0.0049
PDGF Signaling*	0.0105
VEGF Signaling*	0.0158
EGF Signaling^$^	0.0178
**CAN Banff 0 vs. Banff 2&3**	**p-value**
EGF Signaling^$^	0.0145
FGF Signaling*	0.0257
HGF Signaling^$^	0.0347
IGF-1 Signaling*	0.0447
**CAN Banff 1 vs. Banff 2&3**	**p-value**
IGF-1 Signaling*	0.0447

*Significant in two comparisons.

^$^Significant in three or more comparisons.

### MAPK dependent growth factor signaling

Many growth factor pathways are dependent on mitogen-activated protein kinase signaling (MAPK or extracellular-signal-regulated kinase; ERK). We found that LPS-stimulated MAPK, ERK/MAPK and p38 signaling were only significantly differentially expressed in the analysis of Banff 0 vs. Banff 1, 2&3 CAN/IFTA. It is worth noting that MAPK regulates signaling by TGFβ [21-23], VEGF [24-26], IGF-1 [27], and HGF [28-30]. These results further support the analysis of the growth factor pathways above suggesting the same pathways are engaged regardless of the histological severity of CAN/IFTA. The expression of the genes in the TGF, IGF-1 and HGF pathways increased in expression from mild to more severe CAN/IFTA, but the opposite trend was seen with VEGF.

### Integrin pathway and expression

Class comparisons of subjects with and without CAN/IFTA (Banff 0 vs. Banff 1, 2&3) showed that six β-integrin (1, 2, 3, 5, 6, 8) and six α-integrin subunits (v, μ, 1, 2, 6, 9) were upregulated. The canonical integrin signaling pathway was also highly differentially expressed in this comparison (p = 4.1 × 10^-7^). Comparisons of specific integrins significantly upregulated in early CAN/IFTA (Banff 0 vs. Banff 1) revealed differential expression of β subunits 1, 5, 6 and 8, and α subunits v, 1, 6 and μ. With progression from mild to moderate/severe CAN/IFTA (Banff 1 vs. Banff 2&3), the α2, α9, β1, and β6 subunits were upregulated above the levels already present early in CAN/IFTA. The only exception to the up-regulation of integrin expression correlating with CAN/IFTA progression was that integrin α6 was downregulated. All results are summarized in Table 2.

**Table 2:** Integrin subunits differentially expressed between the Banff grades of CAN/IFTA

**B0 vs. 1 + 2 + 3**	**B0 vs. 1**	**B0 vs. 2 + 3**	**B1 vs. 2 + 3**
**β1 (↑1.40)**	**β1 (↑1.33)**	**β1 (↑1.52)**	**β1 (↑1.42)**
**β2(↑1.60)**			
**β3(↑1.25)**			
**β5(↑1.23)**	**β5(↑1.28)**	**β5(↑1.39)**	
**β6(↑3.07)**	**β6(↑2.84)**	**β6(↑6.06)**	**β6(↑2.14)**
**β8(↑1.60)**	**β8(↑1.85)**	**β8(↑1.89)**	
**α1(↑1.45)**	**α1(↑1.64)**	**α1(↑1.69)**	
**α2(↑1.55)**		**α2(↑2.05)**	
**α6(↑1.55)**	**α6(↑1.67)**	**α6(↑1.87)**	**α6(↓1.38)**
**α9(↑1.52)**			**α9(↑1.15)**
**αv(↑1.80)**	**αv(↑1.57)**	**αv(↑2.02)**	
**αμ(↑1.55)**	**αμ(↑1.90)**	**αμ(↑2.53)**	

All p-values are <0.005.

The direction of change is indicated by the arrow and the numbers in brackets represent the magnitude of change for each integrin subunit. All results shown were statistically significant with p values ranging from <1 × 10^-3^ to 10^-7^.

**Figure 1:**
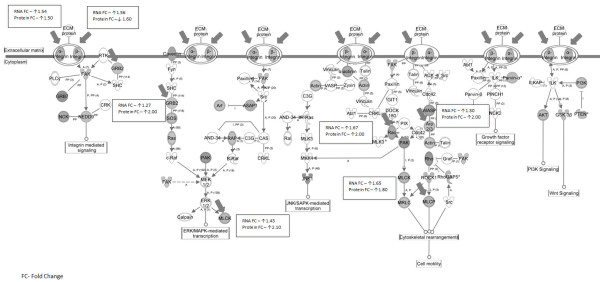
**Integrin pathway molecules identified by the analysis of statistically significant genes between subjects without CAN/IFTA vs. those with CAN/IFTA.** Genes in grey are differentially expressed whereas genes in white are not significantly differentially expressed. It is evident that most genes of the integrin pathway are differentially expressed in CAN/IFTA. The genes that are marked with thick arrows were differentially expressed both at the mRNA level as well as at the protein level by tandem mass spectroscopy proteomics. Protein levels were estimated in this study by using the accumulation of spectral counts for each detected protein as we have previously published [31].

Transcripts for the canonical integrin signaling pathway were also significantly expressed in these latter two comparisons. There are 187 genes in the canonical integrin pathway. Interestingly, only 2 genes, PTEN and PTENP1, were significantly correlated in all three comparisons of CAN/IFTA stages suggesting a potential common signaling mechanism. Thirty-four integrin pathway genes were significantly associated with both Banff 1 and Banff 2&3 CAN/IFTA and 20 genes were only associated with Banff 2&3 (Figure 1). In conclusion, many integrin pathway molecules are highly expressed at an early stage of CAN/IFTA and continue to be expressed during the progression to more severe disease, while another set are linked to only more advanced CAN/IFTA (Table 3).

**Table 3:** An ANOVA analysis of integrin pathway genes significantly differentially expressed between CAN/IFTA Banff 0 (no CAN/IFTA), Banff 1 (mild CAN/IFTA) and Banff 2,3 (moderate severe CAN/IFTA)

**Gene symbol**	**Description**	**Pairwise significance**
**Integrin subunits**		
ITGA1	integrin, alpha 1	(1, 2), (1, 3)
ITGA2	integrin, alpha 2 (CD49B, alpha 2 subunit of VLA-2 receptor)	(1, 3)
ITGA6	integrin, alpha 6	(1, 2), (1, 3), (2, 3)
ITGA9	integrin, alpha 9	(1, 2), (1, 3)
ITGAD	integrin, alpha D	(2, 1), (3, 1)
ITGAM	integrin, alpha M (complement component 3 receptor 3 subunit)	(1, 2), (1, 3)
ITGAV	integrin, alpha V (vitronectin receptor, alpha polypeptide, antigen CD51)	(1, 2), (1, 3)
ITGB1	integrin, beta 1 (fibronectin receptor, beta polypeptide, )	(1, 2), (1, 3), (2, 3)
ITGB2	integrin, beta 2 (complement component 3 receptor 3 and 4 subunit)	(1, 3)
ITGB3	integrin, beta 3 (platelet glycoprotein IIIa, antigen CD61)	(1, 3)
ITGB5	integrin, beta 5	(1, 2), (1, 3)
ITGB6	integrin, beta 6	(1, 2), (1, 3), (2, 3)
ITGB8	integrin, beta 8	(1, 2), (1, 3)
**Integrin signaling pathway molecules associated with all grades of CAN**		
PTEN	phosphatase and tensin homolog	(1, 2), (1, 3), (2, 3)
PTENP1	phosphatase and tensin homolog (mutated in multiple advanced cancers 1), pseudogene 1	(1, 2), (1, 3), (2, 3)
**Integrin signaling pathway molecules associated with mild and moderate CAN but cannot distinguish mild from moderate CAN**		
ACTB	actin, beta	(1, 2), (1, 3)
ACTR2	ARP2 actin-related protein 2 homolog (yeast)	(1, 2), (1, 3)
ACTR3	ARP3 actin-related protein 3 homolog (yeast)	(1, 2), (1, 3)
ACTR3B	ARP3 actin-related protein 3 homolog B (yeast)	(1, 2), (1, 3)
ARF4	ADP-ribosylation factor 4	(1, 2), (1, 3)
ARF6	ADP-ribosylation factor 6	(1, 2), (1, 3)
ARPC1A	actin related protein 2/3 complex, subunit 1A, 41 kDa	(1, 2), (1, 3)
ARPC2	actin related protein 2/3 complex, subunit 2, 34 kDa	(1, 2), (1, 3)
ARPC4	actin related protein 2/3 complex, subunit 4, 20 kDa	(2, 1), (3, 1)
ARPC5	actin related protein 2/3 complex, subunit 5, 16 kDa	(1, 2), (1, 3)
CAPN10	calpain 10	(2, 1), (3, 1)
CAPN6	calpain 6	(2, 1), (3, 1)
CRKL	v-crk sarcoma virus CT10 oncogene homolog (avian)-like	(1, 2), (1, 3)
GRB2	growth factor receptor-bound protein 2	(1, 2), (1, 3)
KRAS	v-Ki-ras2 Kirsten rat sarcoma viral oncogene homolog	(1, 2), (1, 3)
MYLK	myosin, light chain kinase	(1, 2), (1, 3)
NRAS	neuroblastoma RAS viral (v-ras) oncogene homolog	(1, 2), (1, 3)
PAK2	p21 (CDKN1A)-activated kinase 2	(1, 2), (1, 3)
PAK7	p21(CDKN1A)-activated kinase 7	(2, 1), (3, 1)
PIK3C2A	phosphoinositide-3-kinase, class 2, alpha polypeptide	(1, 2), (1, 3)
PIK3C3	phosphoinositide-3-kinase, class 3	(1, 2), (1, 3)
PIK3CA	phosphoinositide-3-kinase, catalytic, alpha polypeptide	(1, 2), (1, 3)
PIK3CB	phosphoinositide-3-kinase, catalytic, beta polypeptide	(1, 2), (1, 3)
PIK3R3	phosphoinositide-3-kinase, regulatory subunit 3 (p55, gamma)	(1, 2), (1, 3)
PPP1CB	protein phosphatase 1, catalytic subunit, beta isoform	(1, 2), (1, 3)
PPP1CC	protein phosphatase 1, catalytic subunit, gamma isoform	(1, 2), (1, 3)
PPP1R12A	protein phosphatase 1, regulatory (inhibitor) subunit 12A	(1, 2), (1, 3)
ROCK1	Rho-associated, coiled-coil containing protein kinase 1	(1, 2), (1, 3)
RRAS2	related RAS viral (r-ras) oncogene homolog 2	(1, 2), (1, 3)
SOS2	son of sevenless homolog 2 (Drosophila)	(2, 1), (3, 1)
TLN1	talin 1	(2, 1), (3, 1)
TSPAN3	tetraspanin 3	(2, 1), (3, 1)
TTN	titin	(2, 1), (3, 1)
WIPF1	WAS/WASL interacting protein family, member 1	(1, 2), (1, 3)
**Integrin signaling pathway molecules specifically associated with moderate CAN**		
ARF1	ADP-ribosylation factor 1	(1, 3)
ARPC1B	actin related protein 2/3 complex, subunit 1B, 41 kDa	(1, 3)
ARPC5L	actin related protein 2/3 complex, subunit 5-like	(1, 3)
CAPN7	calpain 7	(1, 3)
GIT1	G protein-coupled receptor kinase interactor 1	(3, 1)
ILK	integrin-linked kinase	(3, 1)
ITGB3BP	integrin beta 3 binding protein (beta3-endonexin)	(1, 3)
MLCK	MLCK protein	(3, 1)
NCK2	NCK adaptor protein 2	(1, 3)
PAK3	p21 (CDKN1A)-activated kinase 3	(1, 3)
PIK3C2B	phosphoinositide-3-kinase, class 2, beta polypeptide	(1, 3)
PIK3CD	phosphoinositide-3-kinase, catalytic, delta polypeptide	(1, 3)
PIK3CG	phosphoinositide-3-kinase, catalytic, gamma polypeptide	(3, 1)
PIK3R1	phosphoinositide-3-kinase, regulatory subunit 1 (p85 alpha)	(1, 3)
PPP1CA	protein phosphatase 1, catalytic subunit, alpha isoform	(1, 3)
PPP1R12B	protein phosphatase 1, regulatory (inhibitor) subunit 12B	(3, 1)
SOS1	son of sevenless homolog 1 (Drosophila)	(3, 1)
TSPAN4	tetraspanin 4	(1, 3)
TSPAN5	tetraspanin 5	(1, 3)
WASL	Wiskott-Aldrich syndrome-like	(3, 1)

Numbers in brackets represent the pair-wise comparison significance (p < 0.005) where 1 = Banff 0, 2 = Banff 1 and 3 = Banff 2,3.

To give some context with regards to the most significant pathways associated with CAN/IFTA progression from the current study we looked at the integrin, IGF and EGF signaling pathways and their regulation with respect to the progression of CAN/IFTA. For the integrin pathway, we observed that in the Banff 0 vs. 1 comparison all differentially expressed genes in the pathway were upregulated in the Banff 1 patients. When we compared this to the expression of the same genes in the Banff 1 vs. Banff 2,3 patients some of the genes like TLN, JNK and CPN were downregulated in the Banff 2,3 patients. Similar changes were seen in both the HGF and the IGF-1 pathways with PKC and JNK (HGF pathway) being downregulated in the Banff 2,3 when compared to Banff 1, and JNK (EGF pathway) was downregulated in Banff 2,3 when compared to Banff 1, respectively. These changes suggest that some of these genes need not be overexpressed and that their downregulation in the Banff 2,3 group when compared to Banff 1 could possibly be a mechanism that promotes CAN/IFTA progression.

### Proteogenomic analysis of tissue biopsies

In order to validate the results of this gene transcription profiling and functional pathway mapping at the protein level, we turned to our previously published analysis of CAN/IFTA biopsies using shotgun tandem mass spectrometry proteomics [31]. Of the 187 integrin pathway molecules, 25 (14%) of them were identified as significantly differentially expressed by proteomics (SD5) (Additional file 5). In this context it is important to note that we had already identified the whole integrin pathway and specifically integrin β1 protein as a key player in CAN/IFTA progression. In addition, proteins of the IGF-1 pathway were also significant in the progression from mild to moderate/severe CAN/IFTA (Banff 1 vs. Banff 2,3; p = 0.02) and with advanced CAN/IFTA (Banff 0 vs. Banff 2,3; p = 0.004) at the transcript level, which was similar to what we found at the protein level with the IGF-1 pathway being upregulated in the Banff 2,3 subjects, with tandem mass spectrometry proteomics. Finally, in the development of mild CAN/IFTA (Banff 0 vs. Banff 1), the VEGF and EGF pathways were significant (p = 0.02 and 0.05, respectively) by both transcript and protein.

### Drug target and biomarker analysis

We used a new module in IPA based on literature and public drug target databases to determine how many of the molecules we identified by gene expression profiling in CAN/IFTA are targeted by known drugs (SD6) (Additional file 6). Of the 4109 differentially expressed genes between Banff 0 vs. All CAN/IFTA, 129 genes are targets of known drugs. In the differentially expressed growth factor pathways (HGF, VEGF, EGF, IGF-1, TGF-β and PDGF), 10 of the 78 molecules we identified are targets of known drugs. In the integrin pathway, there are only 2 genes known to be targets of drugs (ITGB5 and ITGAV) and both are differentially expressed in our data. We also used Ingenuity to identify genes that are known or potential biomarkers defined in the literature (SD7) Additional file ( 7). For example, of the 78 molecules associated with growth factor signaling, 34 (44%) of the genes are biomarker candidates and 368 of the total 4109 differentially expressed genes have been described as potential biomarkers in the literature in various models of disease.

The advantages of combining information from multiple existing microarray studies in a meta-analysis are a larger sample size, minimization of center-specific effects, and the application of a single data analysis pipeline. Meta-analyses also represent savings in time and costs by making comprehensive use of already available data. The present study represents a mechanism-focused, bioinformatics-driven meta-analysis of gene expression in kidney transplant biopsies with CAN/IFTA. We demonstrate that the differential activation and expression of specific growth factors, integrins and signaling pathways distinguishes the different stages of CAN/IFTA and correlate with progression of disease.

In our data, VEGF and PDGF signaling pathways were significantly upregulated in early CAN/IFTA but were not upregulated in biopsies with moderate/severe CAN/IFTA. This is consistent with an increased expression of VEGF in interstitial cells and arteries developing intimal and adventitial fibrosis in kidneys undergoing vascular rejection [32]. In contrast, only differential expression of IGF-1 signaling correlated with the progression from mild (Banff 1) to moderate/severe CAN/IFTA (Banff 2&3). Perhaps purposeful inhibition of IGF-1 signaling might be a candidate for therapy once CAN/IFTA is already present. However, these results also suggest that most of the growth factor pathways driving CAN/IFTA are present early in the course and not changing significantly as it progresses. It is worth commenting that these results also suggest caution that once patients present with early CAN/IFTA (e.g. protocol biopsy), there is a high likelihood that it will eventually progress.

Interestingly, TGFβ was only differentially expressed when comparing healthy transplants (Banff 0) to those with any grade of CAN/IFTA. However, TGFβ expression does not distinguish between mild and moderate/severe disease. Evidence for the early activation of TGFβ has been shown in a number of studies as early as 2–6 months and is often reviewed as a critical growth factor for interstitial fibrosis [33,34]. A pediatric study showed that relatively early TGFβ expression in grafts 100 days after transplantation correlates with decreased long-term graft function and increased graft fibrosis at 3 years [35]. siRNAs directed against the TGFβ receptor improved renal fibrosis in a mouse model of interstitial nephritis [36]. Our analysis is consistent with these studies in identifying differential TGFβ pathway expression in biopsies with CAN/IFTA compared to healthy transplant biopsies. However, the fact that there is no further increase in TGFβ gene expression despite increasing severity of disease suggests that other pathways mediate progressive fibrosis and tubular atrophy.

The HGF and FGF signaling pathways were significantly upregulated in early CAN/IFTA, but unlike TGFβ were further upregulated in biopsies of moderate/severe CAN/IFTA. HGF, a member of the FGF family, is known to mediate the repair and maintenance of the kidney epithelium. HGF is nephron-protective [37] and suppresses interstitial fibrosis in a rat model of obstructive nephropathy [38]. Exogenous administration of HGF has been shown in a rat model of CAN to prevent the progression of IFTA [39]. Thus, one possibility for our results in these human patients is that up-regulation of the intrinsic HGF pathway acts as a compensatory mechanism protecting the progression of chronic injury and fibrosis. It adds caution to the tendency of advocating blockade of any pathway found in any pathological condition.

FGF is known to stimulate endothelial cell proliferation, degrade ECM and interact with VEGF [40]. *In situ* hybridization and immunohistochemical analysis showed that mRNA for FGF-1 and its high-affinity receptors were increased in the tubular epithelium, inflammatory cell infiltrates, and neovascular structures of kidney transplant patients who underwent nephrectomy after graft loss for what the authors called chronic rejection [41]. Our results are consistent with this role for FGF-1 in biopsy-proven CAN/IFTA.

As already noted, VEGF and PDGF signaling pathways were significantly upregulated with mild CAN/IFTA, but not further upregulated with moderate/severe CAN/IFTA. In a different set of transplant biopsies done several years ago, we also demonstrated the up-regulation of PDGF with CAN/IFTA [42]. Thus, it may be that VEGF and PDGF signaling is important early in the establishment of chronic allograft injury, perhaps by amplifying the injury driven by FGF-1.

The MAPK-dependent growth factor signaling pathways, ERK and p38, were significantly differentially expressed in our study between well functioning transplants vs. any grade of CAN/IFTA. In rat models, the inhibition of p38 MAPK by treatment with a specific p38 MAPK inhibitor resulted in reduced CAN/IFTA with preserved renal function and survival [43,44].

We also show that specific integrins are upregulated in the progression of CAN/IFTA (Table 2) and these results correlate with the parallel up-regulation of the canonical integrin signaling pathway (Figure 1). The integrin subunits β1, β6 and α6 were consistently and significantly differentially expressed in all comparisons**.** An earlier study in rats demonstrated that blockade of interactions between α4β1integrin and fibronectin prevents the development of chronic rejection in cardiac allografts [45]. However, we note that our kidney results do not demonstrate differential expression of the α4 chain. One explanation is that the β1 subunit regulates the assembly of the α4β1integrin in kidneys. A study of integrin expression in allograft rejection showed that αvβ6 was involved in the re-epithelialization of healing isografts in an animal model of chronic progressive lung allograft rejection [46]. In two other studies, mice with bleomycin-induced pulmonary fibrosis were treated with an anti-αvβ6 monoclonal antibody [47,48]. Treated animals showed reduced fibrosis and these results were confirmed using transgenic and knock-out animals. In a published meta-analysis of human kidney transplant biopsies, 309 genes were identified as associated with CAN/IFTA [14]. We identified 47 genes from this set in the present analysis including the α6 chain. Thus, these many lines of evidence converge to identify roles for several specific growth factors and integrin adhesion pathways in chronic kidney transplant injury.

Our analysis of the correlation of the genomic findings with our previously published proteomic work clearly identified the integrin pathway (Figure 1) and the IGF-1 pathway as being differentially regulated and as pathways that specifically affect CAN/IFTA progression. This is very relevant because both these pathways are validated both at the mRNA as well as the protein level and could potentially be the highest priority pathways to address with animal models to study specific mechanisms of CAN/IFTA.

Finally, we used a new module in Ingenuity Pathway Analysis to search for drug targets in our differentially expressed genes and pathways (SD6). For example, in the context of the integrins, there are three drugs that target the integrin αv subunit, two antibodies and one cyclic RGD pentapeptide and the pentapeptide also targets another differentially expressed integrin subunit, β5 [49]. Ten of 78 molecules involved in known growth factor signaling pathways (HGF, VEGF, EGF, IGF-1, TGF-β, PDGF) were targets of over 30 different drugs. Many of these drugs are already FDA-approved while others are in various phases of clinical trials for cancer, hypertension and diabetes, including specific kinase inhibitors, receptor signaling blockers and monoclonal antibodies. The potential of using bioinformatic tools to integrate public data with clinical gene profiling results and identify drug targets or candidate biomarkers is still largely untested. However, two recent studies from Stanford used a similar approach to identify drug targets for cancer, one of which they validated, or search for transplant biomarkers [50,51].

We would also like to draw the attention of the readers to some of the limitations of our study. First, even though we used 84 samples to perform a meta-analysis and despite the fact that we were adequately powered to detect gene expression changes greater than two-fold, the findings of the current study need to be validated in a larger cohort of samples, especially of the different subtypes of CAN before it can be generalized. Similarly, we cannot discount the effect of different immunosuppressive drug regimens and levels on the gene expression, though all the studied patients were on a primary calcineurin inhibitor protocol. The correlations between transcript levels and protein levels have been shown to be relatively poor in earlier studies [52,53]; however several recent studies have shown that these correlations are a lot better than previously shown due to the technical advancements in protein detection by the latest mass spectrometry technologies [54,55]. Our current study used tandem mass spectrometry proteomics data from an earlier study of ours using liquid chromatography coupled with an ion trap instrument (MudPIT protocol for LC/MS/MS) [31]. Newer technologies have significantly enhanced mass detection accuracies and targeted proteomics using alternative mass spectrometry technologies such as multiple single reaction monitoring (MSRM) and triple quadropole TOF instruments will now allow investigators to more accurately identify and even quantify protein level changes for better correlations with gene expression levels profiled in the same samples. The importance of the present work is to establish a clear set of candidate genes and functional pathways that would merit the next stage of experimental efforts to validate these with proteomics.

## Conclusions

Putting the clinical potential of these drug candidate and biomarker correlations into perspective, there are multiple lines of published scientific data that the growth factor signaling pathways we describe here are highly likely to be linked mechanistically to the development and/or progression of CAN/IFTA. Clearly, we cannot determine a root cause of CAN/IFTA simply by gene profiling of biopsies. However, we are describing evidence for multiple and powerful tissue injury and remodeling pathways linked to growth factors and integrin adhesion molecules. The initiation and progression of chronic allograft injury could be driven by immune-mediated rejection through multiple mechanisms and impacted upon by drug toxicities and concomitant medical problems. Regardless, the possibility raised here is that the integrins and growth factors identified in this study are attractive candidates for developing animal models to study the effects of their inhibition/activation. Such work could be the starting point for translational studies into how these molecules modulate signaling in patients with early CAN/IFTA could be a successful way of mitigating the damage caused by whatever the underlying primary mechanism may be and the large number of known drug candidates available is encouraging.

## Methods

### Microarray analysis of tissue biopsies

DNA Microarray CEL files from 84 kidney transplant biopsy samples were studied. We processed 33 samples (24 CAN and 9 Banff0), and the others were from two independent studies (GSE7392 (16 CAN and 14 Banff0) [17] and GSE9493 (16 CAN) [18,19]) from the NIH NCBI GEO repository. The clinical characteristics of the 33 samples processed by us are given in Table 4. All samples were obtained as part of the Transplant Genomics Collaborative Group (TGCG) and approved by the review boards of the participating transplant centers (Scripps Green Hospital, Cleveland Clinic Foundation, Mendez National Institute for Transplantation, Mayo Clinic Arizona and U Colorado Health Sciences). All samples were hybridized on Affymetrix HG-U133 Plus 2.0 Arrays to avoid cross-platform comparisons. We used ComBat, a software that uses Bayes frameworks for adjusting data for batch effects that is robust to outliers in small sample sizes and performs comparable to existing methods for large samples [56]. BRB Array Tools (version 3.8.1) was used to perform class comparisons between classes (p value <0.005 defined as significant after multiple testing adjustments. The resulting FDR ranges reported for all but one comparison were from 1×10^-7^ to 6%. The only exception to this FDR range was the comparison done between early CAN (Banff1) vs. moderate to severe CAN (Banff2,3) where the FDRs ranged from 14 to 19%, consistent with the conclusion that the identified gene changes were much less robust for this comparison. Details on the microarray data processing have been previously described [57]. Pathway, network and drug target analysis was performed using Ingenuity Pathway Analysis (IPA). Pathway significance was determined using a right-tailed Fisher’s Exact Test, where only over-represented functions or pathways are considered significant at a p value ≤0.05.

**Table 4:** Clinical characteristics for the 33 samples that were analyzed with microarrays in our laboratory

	**Banff0**	**CAN**	Significance*
**Subject Numbers**	9	24	
**Recipient Age ± SD**^**‡**^	49.7 ± 12.9	45.7 ± 13.1	NS^^^
**% Female Recipients**	66.7	45.8	NS
**% Recipient African American**	0	8.33	NS
**% Pre-tx Type II Diabetes**	25	17.4	NS
**% PRA > 20**	0	9.5	NS
**HLA Mismatch ± SD**	2.3 ± 1.3	2.6 ± 3.2	NS
**% Deceased Donor**	66.7	69.6	NS
**Donor Age ± SD**	31.4 ± 13.6	41.6 ± 14.7	NS
**% Female Donors**	33.3	41.6	NS
**% Donor African American**	0	6.7	NS
**% Induction**	66.7	87.5	NS
**Serum Creatinine ± SD**	3.07 ± 1.2	1.78 ± 1.1	0.01
**Time to Biopsy (Days) ± SD**	691 ± 550	2009 ± 1367	0.0004
**% Calcineurin Inhibitors**	87.5	75	NS
**% Mycophenolic Acid Derivatives**	55.6	70.8	NS
**% Oral Steroids**	44.5	100	0.0005
**C4d Positive Staining (%)**	0	13	NS

* Significance for all comparisons were determined with paired Students t-test for pair-wise comparisons of data with Standard Deviations and for dichotomous data comparisons by Chi-Square.

^**‡**^ SD = standard deviation from the mean.

^ NS = not significant (p ≥ 0.05).

### Proteogenomic analysis of tissue biopsies

All proteomic data comparisons were made using our previously published study that revealed about 1400 proteins with unique expression profiles tracing the progression from normal transplant biopsies to increasingly severe grades of CAN/IFTA [31].

### Consent

Written informed consent was obtained from all the patients in this study for publication of this manuscript.

## Competing interests

The author(s) declare that they have no competing interests.

## Authors’ contributions

AD: Participated in research design, participated in the writing of the paper and participated in data analysis. ER: Participated in the performance of the research. TM: Participated in the performance of the research. SRH: Participated in research design and participated in the performance of the research. DRS: Participated in research design and participated in the writing of the paper. SMK: Participated in research design, participated in the writing of the paper and participated in data analysis. All authors read and approved the final manuscript.

## Supplementary Material

Additional file 1 Banff 0 vs. All CAN. Click here for file

Additional file 2 CAN Banff 0 vs Banff 1. Click here for file

Additional file 3 CAN Banff 0 vs. Banff 2,3. Click here for file

Additional file 4 CAN Banff 1 vs Banff 2,3. Click here for file

Additional file 5 25 integrin pathway molecules identified as significantly differentially expressed by proteomics. Click here for file

Additional file 6 Drug Targets - Healthy transplants vs. CAN/IFTA subjects (129/4109 molecules). Click here for file

Additional file 7 Growth Factor Signaling Pathway biomarker Candidates (34/78 molecules). Click here for file
